# Seizure Clusters: Morbidity and Mortality

**DOI:** 10.3389/fneur.2021.636045

**Published:** 2021-02-16

**Authors:** Kristie Bauman, Orrin Devinsky

**Affiliations:** Department of Neurology, NYU Grossman School of Medicine and NYU Langone Comprehensive Epilepsy Center, New York, NY, United States

**Keywords:** seizure, seizure cluster, SUDEP (sudden unexplained death in epilepsy), morbidity, mortality

## Abstract

Seizure clusters, an intermediate between single seizure and status epilepticus, are associated with morbidity, impaired quality of life, and premature mortality. The relationship between seizure clusters and sudden unexplained death in epilepsy (SUDEP) is poorly understood. Here, we define seizure clusters; review comorbid psychiatric disorders and memory deficits associated with seizure clusters; and review cases of witnessed SUDEP for which seizure frequency prior to death is available. Patients with a history of seizure clusters have a 2.5 fold increased risk for SUDEP, and one third of patients with monitored in hospital SUDEP experienced a cluster of generalized tonic clonic seizures prior to death. Understanding the effects of seizure frequency and duration on SUDEP risk could yield new insights in SUDEP pathophysiology and new targets for intervention.

## Introduction

Seizure duration forms a continuous range from brief single seizures to refractory status epilepticus with seizure clusters as intermediate, whose durations, number of discrete events and severities vary widely. The border between single seizure, seizure cluster and status epilepticus is obscured by overlapping biological phenomena and the lack of a precise, readily quantified, or widely accepted definition of a seizure cluster. Seizure clusters are typically defined by the number of seizures over a specific interval or as an increased seizure frequency above baseline recognizable by patient or family (1–5). For many patients, seizure clusters impair quality of life and increase morbidity (hospitalization, status epilepticus, postictal psychosis), and mortality (6–11).

The relationship between seizure clusters and sudden unexplained death in epilepsy (SUDEP) remains poorly defined. The borders between SUDEP, death following seizure clusters, and death from status epilepticus are unclear. Terminologies vary widely among and between epileptologists focused on clinical care and those focused on SUDEP research, and forensic pathologists/medical examiners. Because >99% of SUDEPs occur out of hospital and ~90% are unwitnessed, the presence, types and durations of seizures preceding death are poorly characterized (12, 13). Current SUDEP criteria exclude documented status epilepticus, although in individual cases, it is often impossible to determine if status preceded death. Here, we examine the definitions of seizure clusters, and relationships between premature death and seizure frequency and between seizure clusters and SUDEP.

## What Are Seizure Clusters?

Seizure clusters lack a uniformly accepted clinical (see Table 1) or statistical definition (14, 15). Many studies have proposed clinical definitions of seizure clusters as a number of seizures over a unit of time. This is founded on the finding that seizures that recur within 8 h are more likely to come from a concordant focus and are therefore not independent of each other (7, 16). Thus, many define seizure clusters as > 3 seizures over 24 h since the average interictal period is ≤ 8 h (1, 3–5, 8, 9, 11, 17, 18). Others have broadened the definition to 2 or more seizures in 24 h to allow easier patient recall of seizure clusters (19, 20). More recent studies have both shortened the time interval and decreased the number of seizures required to define a cluster to > 2 seizures in 6 h (21, 22). This change encourages earlier recognition of seizure clusters and earlier use of rescue medication but misses clusters which occur over a longer period of time.

**Table 1:** Clinical definitions of seizure clusters.

**References**	**Time Interval**	**Seizure Number and Type**
Di Gennaro et al. (1) Noe and Drazkowski (18) Haut et al. (8) Rose et al. (4) Haut et al. (3) Yen et al. (5) Haut et al. (9)	24 h	> 3 focal impaired awareness seizures
Martinez et al. (17) Sillanpää and Schmidt (11)	24 h	> 3 focal or generalized seizures (excluding absence and myoclonic seizures)
Penovich et al. (20) Fisher et al. (19)	24 h	> 2 seizures of any type
Detyniecki et al. (22) Detyniecki et al. (21)	6 h	> 2 seizures of any type
Di Gennaro et al. (1) Noe and Drazkowski (18) Rose et al. (4)	4 h	> 3 focal impaired awareness seizures

It is important to also consider which seizure types should be counted when defining seizure clusters. Seizure severity is one factor to consider as some seizure types may have a greater effect on quality of life, morbidity, and mortality. Many studies in presurgical patients have therefore included only focal impaired awareness and focal to bilateral tonic clonic seizures in their definitions of seizure clusters (1, 3–5, 8, 9, 18). While some suggest including all seizure types for ease of patient recall and reporting (19), this may overestimate the prevalence of clinically relevant seizure clusters if seizures which typically occur at high frequencies are included. Many therefore exclude absence and myoclonic seizures (11, 17). Similarly, many excluded generalized epilepsy syndromes which are associated with a high seizure burden such as West Syndrome and Lennox Gastaut since typical seizure frequency in these patients often far exceeds the numeric seizure threshold for clustering (11). However, these patients can still have a clinically relevant increase in seizure frequency above baseline which should also be considered seizure clusters.

Statistical definitions of seizure clusters may be more applicable in some scenarios than definitions which focus on seizure number and interval alone. Bauer and Burr (23) proposed one of the most inclusive statistical definitions for clusters: “those time periods during which seizure occurrence is significantly increased compared to the rate expected from an individual mean.” Others defined clusters as a threefold increase in the patient's typical seizure frequency over a three day period (24).

Both clinical and statistical definitions fail to capture clinically relevant clusters in subsets of patients. A clinical definition > 2 seizures in 6 h encourages earlier use of rescue medication and may decrease incidence of adverse events due to clustering, but may miss patients whose clusters occur over longer periods of time. A definition of 3 seizures in a 24 h period captures these patients, but may overestimate seizure clusters in patients with high seizure frequency. On the other end of the spectrum, statistical definition of clusters such as a three fold increase in seizure frequency overestimates clusters in patients with low seizure frequency, such as a single breakthrough seizure in a previously seizure free patient. These definitions have the potential to overestimate seizure frequency due to the failure to account for random variations in seizure frequency. More recently, the algorithm *ClusterCalc* has been proposed to improve sensitivity and specificity in identifying seizure clusters by accounting for random variation (25). This algorithm demonstrated that 37–59% of clusters identified by traditional definitions may have occurred due to chance (25).

Seizure clusters also reflect an ongoing debate whether “seizures beget seizures” (26). Although this maxim is often considered from the perspective of seizures dispersed more widely in time (e.g., a boy has convulsive seizures at age 8, 11, 12, and 12.5—the shortening interval possibly reflecting another maxim), it is also consistent with recurrent seizures over a brief interval (i.e., seizure cluster). Both interpretations are also consistent with Hebb's maxim that “neurons that fire together wire together” (27).

The prevalence of seizure clusters varies by population. Among inpatients with drug resistant epilepsy undergoing anti-seizure medication (ASM) withdrawal for pre-surgical evaluations, 39 to 61% experience seizure clusters over a 24 h period during a study lasting 2–12 days (1, 3, 4, 9). Among outpatients, seizure clusters are less frequent, occurring in 22 to 34% of patients over years (11, 21, 28, 29). However, after excluding patients who are seizure free, the rates of clustering among those with active epilepsy is higher at 52% and even higher at 71% among those with > 4 seizures per year (21).

Seizure clusters are associated with significant morbidity. Depending on the individual, the severity and frequency of seizures within a cluster, and other factors, seizure clusters may predispose to acute or more long-lasting psychiatric, cognitive or physical disorders. Postictal psychosis is more common after clustering. Case series and case- control cohort studies of patients with postictal psychosis showed a trend toward high rates of seizure clustering before postictal psychosis, though this did not reach statistical significance in these underpowered studies (6, 10). Clusters may cause long term disability through the development of interictal psychosis. In one case series, 13.4% of patients with postictal psychosis developed interictal psychosis (30). Seizure clusters may drive changes in neuronal networks that lower the threshold for psychosis and depress normal neuronal function over time (30). Despite their association with psychosis, seizure clusters are not associated with higher rates of anxiety or depression (8).

The effect of seizure clusters on memory is not well defined as we lack well-powered prospective case-control studies. Our data cannot distinguish the direct adverse effects of clusters from progression of epileptogenesis or the underlying disorder, medication effects and other factors (e.g., head injury from seizures, depression). While postictal and interictal psychosis are associated with memory and other cognitive impairments, seizure clusters could theoretically have beneficial cognitive effects by increasing the inter-seizure interval and allowing longer periods of brain “rest” with seizure freedom.

Compared to those with weekly, or daily seizures (*N* = 32), those with seizure clusters with seizure free intervals of at least 2 weeks (*N* = 8) had significantly higher IQ, arithmetic, and reading comprehension scores (31). However, this study did not quantify total seizure number and cannot distinguish the effect of seizure frequency or seizure number on cognition.

Patients with seizure clusters have higher rates of several adverse outcomes, including failure to achieve seizure remission during treatment (i.e., treatment-resistant epilepsy) (11), high seizure frequencies (8, 11) and more frequent hospitalizations (8). Seizure clusters also affect many aspects of quality of life with a majority of patients reporting negative effects on their career, independence, ability to drive, extracurricular, and social activities, and ability to travel (20). Evidence supports and refutes an association between seizure clusters and status epilepticus (3, 8, 9). The burden of prolonged seizures and status epilepticus among seizure cluster patients is likely underestimated since many patients live alone and have no recall for clusters or status. Further, changing definitions of clusters and status epilepticus (e.g., 10 vs. 30 min) limits comparisons across studies. Seizure clusters affect large portions of the epilepsy population and likely contribute directly to some deaths.

## Seizure Clusters and Mortality

In one population study, people with epilepsy had an 11 fold higher odds of premature death compared to matched general population controls (32). The risk of sudden unexplained death is even higher. A Danish cohort of children and young adults with epilepsy had a 27 fold higher risk of sudden unexplained death, which remained elevated at 16 times higher risk even after adjusting for comorbidities associated with epilepsy (33). Although many deaths result from the underlying cause of epilepsy (e.g., neoplasms, stroke, perinatal disorders), 15.8% were caused by external causes. Individuals with epilepsy were more likely to die from suicide, assault, motor vehicle accidents, and non-vehicle accidents such as falls and drowning (32). This risk accrues with increasing seizure frequency, seizure clustering, and time since epilepsy diagnosis. In a Finnish cohort of childhood onset epilepsy patients followed for ~40 years found that patients with seizure clusters had a 3.5-fold greater frequency of premature death (42%) than those without seizure clusters (14%) (11). This risk may be modifiable as patients with seizure clusters who subsequently had better seizure control did not have an increased risk of death (11). Seizure control strongly influenced SUDEP risk. Adult patients with childhood onset epilepsy with poor seizure control had a 5-fold increased risk of SUDEP (34). However, the relationship between seizure clusters and SUDEP is poorly understood.

## Seizure Clusters and SUDEP

Nearly 90% of SUDEPs are unwitnessed. Our understanding of the relationship between SUDEP and seizure duration and frequency is limited to witnessed out of hospital SUDEPs and SUDEPs which recorded by video EEG. While status epilepticus cases are excluded from SUDEP, it is difficult and often impossible to differentiate seizure clusters during sleep from status epilepticus without video EEG since observers cannot distinguish ongoing nonconvulsive seizures, postictal sedation, and normal sleep. Here, we review the existing case series and case reports of witnessed SUDEP and the seizure frequency prior to death as available.

A series of 15 witnessed out-of-hospital SUDEP found that 12 were preceded by GTC, one by focal seizure, and two by post-ictal state due to seizure of unknown semiology (35). While status epilepticus cases (seizures >30 min or multiple seizures without return to baseline) were excluded, the identification of seizure clusters or status epilepticus was severely limited in this population since most patients were found in bed (35). Thus, recognition of seizure could be delayed and family members or caregivers may only observe the seizure after they awaken (e.g., at 6 a.m.) or when they hear noises associated with a seizure, which again may correspond with lighter stages of sleep. Thus, even in witnessed cases, the true seizure frequency or duration during the hours before death is rarely known with certainty. While seizure number in the 24 h before death could identify seizure cluster cases, this data is limited by recall bias and memory lapses due to delays in interviewing family members after SUDEP occurred.

Other studies used historical seizure frequency to characterize seizure frequency before SUDEP. In the Finnish pediatric cohort followed prospectively for ~40 years, 22% of patients had >1 lifetime seizure cluster (11). SUDEP was >2.5-fold more frequent among those with seizure clusters than those without (8 vs. 3%), but the small sample size limited statistical power (11). The effect of seizure clusters on SUDEP risk in epileptic encephalopathies is poorly understood. Seizure clusters are common in several developmental epileptic encephalopathies, including Dravet and PCDH19 syndromes, in which clusters are often provoked by fever. Although seizure clusters occur in more than 90% of PCDH19 patients (36), SUDEP is rarely reported. By contrast, patients with Dravet syndrome often have nocturnal clusters of tonic or tonic-clonic seizures, especially between ages 4–11 years of age (37). It is possible that a tendency to more severe seizure clusters in sleep contributes to SUDEP risk in Dravet syndrome, although further study, ideally including a spectrum of developmental epileptic encephalopathies would be helpful in identifying relevant factors.

The MORTality in Epilepsy Monitoring Unit Study (38), reviewed 29 cardiorespiratory arrests in patients observed in epilepsy monitoring units. This landmark study provided important insights into SUDEP pathophysiology. These cases may differ in some ways from out of hospital SUDEP since most had anti-seizure medication withdrawal before death, although the majority of out-of-hospital SUDEPs were found to have subtherapeutic or undetectable anti-seizure medication levels or did not take their last dose of medication, suggesting many underwent medication withdrawal (12, 13, 39). MORTEMUS included 16 cases of definite or probable SUDEP, 2 fatal near SUDEP, 7 non-fatal near SUDEP, and 4 deaths due to other causes (38). Like other studies, nearly all SUDEPs occurred overnight, limiting data in cases where the patient not being monitored when death occurred. All 11 monitored SUDEP cases were preceded by a GTC. Among these cases, 7 cases had only the one terminal GTC in the 12 h preceding death. Of the remaining four cases, two patients had 5 GTCs, one had 4 GTCs, and one had 2 GTCs in the 12 h preceding death. Unfortunately, seizure duration was not reported, and while no deaths resulted from status epilepticus, the definition of status epilepticus was not explicitly defined and series spanned a period in which diagnostic criteria for status epilepticus changed. Further, most SUDEPs occurred in sleep, making it uncertain if patients returned to baseline between seizures, which makes it impossible to distinguish a cluster from status epilepticus. Also, non-GTCs were not quantified and the number of patients with clusters of focal and generalized seizures before death is unknown. In MORTEMUS, 36% of patients had clusters of 2 or more GTCs in the 24 h prior to death, but the true prevalence of seizure clusters with focal unaware and focal aware seizures is likely higher (38).

## Conclusion

Although epilepsy is associated with morbidity and mortality, these risks appear to be higher in individuals with a seizure cluster. People with epilepsy have an 11 times higher risk of premature death (32), and patients with seizure clusters are at even higher risk with one cohort finding a 3.5 fold higher risk of premature death in those with seizure clusters compared to those without (11).

Poor seizure control and seizure medication withdrawal are risk factors for SUDEP (12, 13, 34, 39). This could suggest that seizure clusters, which may be more frequent in these groups, are also a risk factor for SUDEP. Patients with a history of seizure clusters during their lifetime had a 2.5 fold increased risk of SUDEP, though this study was underpowered due to small sample size (11). Among cases of monitored in hospital SUDEP, 36% had clusters of GTCs in the 24 h prior to death (38). The rates of focal seizure clusters in this population is unknown, and the prevalence of both focal and generalized seizure clusters prior to SUDEP is likely higher.

The greatest limitation in our understanding of the pathophysiology and effects of seizure clusters is the lack of a consensus definition (14, 15). Existing definitions are often tailored to the patient population being studied: > 2 seizures in 24 h for outpatient seizure diary studies which require patient recall (19, 20), > 2 seizures in 6 h for studies examining efficacy of rescue medications (22), or statistical definitions for those with high seizure frequency such as in Lennox Gastaut Syndrome. While these definitions each have strengths within specific patient populations, the variation limits comparisons of studies and generalizability of findings. The consensus definition for seizure clusters should aim to identify seizure clusters as soon as possible to allow earlier intervention while also striving to not over or underestimate clinically relevant seizure clusters. Algorithms such as *ClusterCalc* identify clusters while accounting for randomness in inter-seizure interval variation and may improve our ability to study clinically significant seizure clusters (25). Further research in this area may help determine when clusters are more likely to self-terminate, continue, or result in status epilepticus or SUDEP and allow targeted early intervention to prevent adverse effects of seizure clusters.

The effect of seizure clusters on SUDEP risk remains poorly understood. Research in this area has been limited by multiple factors: (1) >99% of SUDEPs occur out of hospital, (2) ~90% of SUDEPs are unwitnessed, and (3) recall bias and memory lapses due to delays in interviewing witnesses of SUDEP. While the effect of the first two factors is difficult to overcome, the third may be a target for intervention in future studies. The rise of electronic seizure diaries could offer new insights into seizure frequency and duration in the hours to days prior to SUDEP. Similarly, seizures captured through responsive neurostimulation may be readily quantified, though this population is limited and remains small at this time. Understanding the effects of seizure frequency and duration on SUDEP risk could offer insights in the SUDEP pathophysiology and new targets for intervention.

## Author Contributions

Both authors, KB and OD, contributed on conception, design and drafting the article or revising it critically for important intellectual content. They both approved the final version.

## Conflict of Interest

The authors declare that the research was conducted in the absence of any commercial or financial relationships that could be construed as a potential conflict of interest.

## References

[1] Di Gennaro G Picardi A Sparano A Mascia A Meldolesi GN Grammaldo LG. Seizure clusters and adverse events during pre-surgical video-EEG monitoring with a slow anti-epileptic drug (AED) taper. Clin Neurophysiol. (2012) 123:486–8. 10.1016/j.clinph.2011.08.011 21920813

[2] Haut SR. Seizure clusters: characteristics and treatment. Curr Opin Neurol. (2015) 28:143–50. 10.1097/wco.0000000000000177 25695133

[3] Haut SR Swick C Freeman K Spencer S. Seizure clustering during epilepsy monitoring. Epilepsia. (2002) 43:711–5. 10.1046/j.1528-1157.2002.26401.x 12102673

[4] Rose AB McCabe PH Gilliam FG Smith BJ Boggs JG Ficker DM. Occurrence of seizure clusters and status epilepticus during inpatient video-EEG monitoring. Neurology. (2003) 60:975–8. 10.1212/01.wnl.0000053748.83309.28 12654962

[5] Yen DJ Chen C Shih YH Guo YC Liu LT Yu HY. Antiepileptic drug withdrawal in patients with temporal lobe epilepsy undergoing presurgical video- EEG monitoring. Epilepsia. (2001) 42:251–5. 10.1046/j.1528-1157.2001.15100.x 11240598

[6] Alper K Kuzniecky R Carlson C Barr WB Vorkas CK Patel JG. Postictal psychosis in partial epilepsy: a case-control study. Ann Neurol. (2008) 63:602–10. 10.1002/ana.21341 18481288

[7] Haut SR. Seizure clustering. Epilepsy Behav. (2006) 8:50–5. 10.1016/j.yebeh.2005.08.018 16246629

[8] Haut SR Shinnar S Moshé SL. Seizure clustering: risks and outcomes. Epilepsia. (2005) 46:146–9. 10.1111/j.0013-9580.2005.29004.x 15660781

[9] Haut SR Shinnar S Moshé SL O'Dell C Legatt AD. The association between seizure clustering and convulsive status epilepticus in patients with intractable complex partial seizures. Epilepsia. (1999) 40:1832–4. 10.1111/j.1528-1157.1999.tb01607.x 10612353

[10] Logsdail SJ Toone BK. Post-ictal psychoses. A clinical and phenomenological description. Br J Psychiatry. (1988) 152:246–52. 10.1192/bjp.152.2.246 3167343

[11] Sillanpää M Schmidt D. Seizure clustering during drug treatment affects seizure outcome and mortality of childhood-onset epilepsy. Brain. (2008) 131:938–44. 10.1093/brain/awn037 18310680

[12] Pollanen MS Kodikara S. Sudden unexpected death in epilepsy: a retrospective analysis of 24 adult cases. Forensic Sci Med Pathol. (2012) 8:13–8. 10.1007/s12024-011-9263-4 21800178

[13] Verducci C Hussain F Donner E Moseley BD Buchhalter J Hesdorffer D. SUDEP in the North American SUDEP Registry: the full spectrum of epilepsies. Neurology. (2019) 93:e227–e36. 10.1212/wnl.0000000000007778 31217259 PMC6656646

[14] Gidal B Klein P Hirsch LJ. Seizure clusters, rescue treatments, seizure action plans: Unmet needs and emerging formulations. Epilepsy Behav. (2020) 112:107391. 10.1016/j.yebeh.2020.107391 32898744

[15] Jafarpour S Hirsch LJ Gaínza-Lein M Kellinghaus C Detyniecki K. Seizure cluster: Definition, prevalence, consequences, and management. Seizure. (2019) 68:9–5. 10.1016/j.seizure.2018.05.013 29871784

[16] Haut SR Legatt AD O'Dell C Moshé SL Shinnar S. Seizure lateralization during EEG monitoring in patients with bilateral foci: the cluster effect. Epilepsia. (1997) 38:937–40. 10.1111/j.1528-1157.1997.tb01260.x 9579896

[17] Martinez C Sullivan T Hauser WA. Prevalence of acute repetitive seizures (ARS) in the United Kingdom. Epilepsy Res. (2009) 87:137–43. 10.1016/j.eplepsyres.2009.08.006 19748226

[18] Noe KH Drazkowski JF. Safety of long-term video-electroencephalographic monitoring for evaluation of epilepsy. Mayo Clin Proc. (2009) 84:495–500. 10.1016/s0025-6196(11)60580-6 19483165 PMC2688622

[19] Fisher RS Bartfeld E Cramer JA. Use of an online epilepsy diary to characterize repetitive seizures. Epilepsy Behav. (2015) 47:66–71. 10.1016/j.yebeh.2015.04.022 26046724

[20] Penovich PE Buelow J Steinberg K Sirven J Wheless J. Burden of seizure clusters on patients with epilepsy and caregivers: survey of patient, caregiver, clinician perspectives. Neurologist. (2017) 22:207–14. 10.1097/nrl.0000000000000140 29095321

[21] Detyniecki K O'Bryan J Choezom T Rak G Ma C Zhang S. Prevalence and predictors of seizure clusters: a prospective observational study of adult patients with epilepsy. Epilepsy Behav. (2018) 88:349–56. 10.1016/j.yebeh.2018.09.035 30344026

[22] Detyniecki K Van Ess PJ Sequeira DJ Wheless JW Meng TC Pullman WE. Safety and efficacy of midazolam nasal spray in the outpatient treatment of patients with seizure clusters-a randomized, double-blind, placebo-controlled trial. Epilepsia. (2019) 60:1797–808. 10.1111/epi.15159 31140596 PMC9291143

[23] Bauer J Burr W. Course of chronic focal epilepsy resistant to anticonvulsant treatment. Seizure. (2001) 10:239–46. 10.1053/seiz.2000.0499 11466018

[24] Newmark ME Dubinsky S. The significance of seizure clustering: a review of 343 outpatients in an epilepsy clinic. In: Dreifuss FE editor. Chronopharmacology in Therapy of the Epilepsies. New York, NY: Raven Press (1990). p. 89–103.

[25] Chiang S Haut SR Ferastraoaru V Rao VR Baud MO Theodore WH. Individualizing the definition of seizure clusters based on temporal clustering analysis. Epilepsy Res. (2020) 163:106330. 10.1016/j.eplepsyres.2020.106330 32305858

[26] Gowers WR. Epilepsy and Other Chronic Convulsive Disorders: Their Causes, Symptoms and Treatment. London: JandA Churchill. (1881).

[27] Keysers C Gazzola V. Hebbian learning and predictive mirror neurons for actions, sensations and emotions. Philos Trans R Soc Lond B Biol Sci. (2014) 369:20130175. 10.1098/rstb.2013.0175 24778372 PMC4006178

[28] Bauer J Ghane Y Flügel D Wildt L Stefan H. Etiology, follow-up and therapy of seizure clusters in temporal lobe epilepsy and catamenial epileptic seizures. Schweiz Arch Neurol Psychiatr. (1992) 143:117–34. 1375777

[29] Manford M Fish DR Shorvon SD. An analysis of clinical seizure patterns and their localizing value in frontal and temporal lobe epilepsies. Brain. (1996) 119:17–40. 10.1093/brain/119.1.17 8624679

[30] Tarulli A Devinsky O Alper K. Progression of postictal to interictal psychosis. Epilepsia. (2001) 42:1468–71. 10.1046/j.1528-1157.2001.10701.x 11879351

[31] Smith ML Elliott IM Lach L. Cognitive skills in children with intractable epilepsy: comparison of surgical and nonsurgical candidates. Epilepsia. (2002) 43:631–7. 10.1046/j.1528-1157.2002.26101.x 12060023

[32] Fazel S Wolf A Långström N Newton CR Lichtenstein P. Premature mortality in epilepsy and the role of psychiatric comorbidity: a total population study. Lancet. (2013) 382:1646–54. 10.1016/s0140-6736(13)60899-5 23883699 PMC3899026

[33] Holst AG Winkel BG Risgaard B Nielsen JB Rasmussen PV Haunsø S. Epilepsy and risk of death and sudden unexpected death in the young: a nationwide study. Epilepsia. (2013) 54:1613–20. 10.1111/epi.12328 23895621

[34] Sillanpä M Shinnar S. Long-term mortality in childhood-onset epilepsy. N Engl J Med. (2010) 363:2522–2529. 10.1056/NEJMoa0911610 21175314

[35] Langan Y Nashef L Sander JW. Sudden unexpected death in epilepsy: a series of witnessed deaths. J Neurol Neurosurg Psychiatry. (2000) 68:211–3. 10.1136/jnnp.68.2.211 10644790 PMC1736784

[36] Smith L Singhal N El Achkar CM Truglio G Rosen Sheidley B Sullivan J. PCDH19-related epilepsy is associated with a broad neurodevelopmental spectrum. Epilepsia. (2018) 59:679–89. 10.1111/epi.14003 29377098 PMC6264912

[37] Losito E Kuchenbuch M Chemaly N Laschet J Chiron C Kaminska A. Age-related “Sleep/nocturnal” tonic and tonic clonic seizure clusters are underdiagnosed in patients with Dravet Syndrome. Epilepsy Behav. (2017) 74:33–40. 10.1016/j.yebeh.2017.05.037 28683344

[38] Ryvlin P Nashef L Lhatoo SD Bateman LM Bird J Bleasel A. Incidence and mechanisms of cardiorespiratory arrests in epilepsy monitoring units (MORTEMUS): a retrospective study. Lancet Neurol. (2013) 12:966–77. 10.1016/s1474-4422(13)70214-x 24012372

[39] Ngugi AK Bottomley C Fegan G Chengo E Odhiambo R Bauni E. Premature mortality in active convulsive epilepsy in rural Kenya: causes and associated factors. Neurology. (2014) 82:582–9. 10.1212/wnl.0000000000000123 24443454 PMC3963418

